# Performance of a reflectometric technique for serum alanine aminotransferase determinations

**DOI:** 10.1155/S1463924689000301

**Published:** 1989

**Authors:** J. W. M. Keen

**Affiliations:** Biochemistry Department York District Hospital York UK

## Abstract

The Ames Seralyzer uses solid phase chemistry coupled with reflectance spectroscopy to measure levels of serum alanine aminotransferase (serum ALT). The study reported here was carried out to evaluate the performance of the Seralyzer method compared to conventional analyses carried out on a Coulter Dacos discrete random access analyser using Boehringer (BCL) reagents conforming to SCE specications at 37° C. Clinically acceptable accuracy and precision were obtained. The effect of bilirubin and haemoglobin on the test was investigated. Increased levels of bilirubin in sera did not affect the estimation but the presence of high haemoglobin levels produced significant interference. The overall practicality of the Seralyzer was assessed and the method was found to be particularly suitable for the estimation of urgent or high risk samples in the laboratory or for use by suitably trained personnel in other clinical areas.


Journal of Automatic Chemistry Vol. 11, No. 3 (May–June 1989), pp. 134–136

### Performance of a reflectometric technique for serum alanine aminotransferase determinations

**J. W. M. Keen**
*Biochemistry Department, York District Hospital, York, UK*

*The Ames Seralyzer uses solid phase chemistry coupled with reflectance spectroscopy to measure levels of serum alanine aminotransferase (serum ALT). The study reported here was carried out to evaluate the performance of the Seralyzer method compared to conventional analyses carried out on a Coulter Dacos discrete random access analyser using Boehringer (BCL) reagents conforming to SCE specifications at 37 °C. Clinically acceptable accuracy and precision were obtained. The effect of bilirubin and haemoglobin on the test was investigated. Increased levels of bilirubin in sera did not affect the estimation but the presence of high haemoglobin levels produced significant interference. The overall practicality of the Seralyzer was assessed and the method was found to be particularly suitable for the estimation of urgent or high risk samples in the laboratory or for use by suitably trained personnel in other clinical areas.*

### Introduction

Several discrete analysers have been designed for use in the solid phase. Generally, the principle of operation involves reacting an analyte with a dry chemical matrix incorporating the reagents. This produces a change in the visible or ultraviolet spectrum which is measured using reflectance spectroscopy. The advantages of this kind of technology are many: the single test reaction pads need no preparation, have a long shelf life and the methods use simple standard procedures.

The Seralyzer Blood Chemistry Analyser manufactured by Ames Division (Miles Ltd) was specifically designed to measure reflectance levels using dedicated test strips. The theory and principles of operation of the analyser have been fully described by Zipp and Hornby [1] and Stevens and Newall [2]. The performance of several analytical procedures have been reported, including serum or plasma urea, glucose, urate [2], CK, LDH [3] and AST [4].

This paper describes the evaluation of a new ALT (alanine aminotransferase, European Enzyme No. 2.6.1.2) determination using the Seralyzer. The results obtained are compared with analyses carried out on the same day on a Coulter Dacos discrete random access analyser using Boehringer (BCL) reagents conforming to SCE specifications at 37 °C. The effect of bilirubin and haemoglobin on the test was investigated and the overall practicality assessed.

### Method

The ALT test strip contains a matrix which, in the presence of ALT enzyme, produces a coloured dye complex. The reaction sequence is as follows:

$$\text{Alanine} + \alpha\text{-ketoglutarate} \xrightarrow{\text{ALT}} \text{pyruvate} + \text{glutamate}$$

$$\text{Pyruvate} + \text{phosphate} + O_2 \xrightarrow[\text{thiaminepyrophosphate}]{\text{pyruvate oxidase, } Mg^{2+}} \text{acetylphosphate} + CO_2 + H_2O_2$$

$$H_2O_2 + \text{4-AAP} + \text{DCHBS} \xrightarrow{\text{peroxidase}} \text{quinoneimine dye} + H_2O$$

where 4-AAP = 4-aminoantipyrine and DCHBS = 3,4-dichloro-2-hydroxybenzenesulphonate.

The Seralyzer reflectometer system measures the coloured quinoneimine dye formed on the test strip by monitoring the total change in reflectance at 530 nm at 37 °C over a period of 4 min. The assay requires 30 µl of serum diluted 1 : 3 in water which is pipetted on to the dry reaction pad on the strip. The test strip is immediately inserted into the reflectometer. If the concentration of ALT is greater than 250 IU $l^{-1}$ an over-range code shows on the display. In this event a further 9-fold dilution of the serum is made and the sample is reanalysed.

*Protocol*

Each day, a two-point calibration was performed on the Seralyzer using reconstituted lyophilised calibrator serum. Duplicate analyses were carried out on three control sera (Gilford Normal, Abnormal and Wellcome) and up to 12 patient samples. Daily calibration was for evaluation purposes only: in practice, the calibration is stable and would not be required so frequently.

Samples from the controls and patients were analysed singly on the Dacos analyser for ALT and for bilirubin. A total of 115 clinical samples were analysed.

### Results

Between-batch precision was analysed using the data given by the daily control results. As the data in table 1 indicate, the Seralyzer gave higher mean readings for the Gilford N ($p < 0{\cdot}001$) and lower for Gilford A ($p = 0{\cdot}004$)

0142–0453/89 $3.00 © 1989 Taylor & Francis Ltd.

*Table 1. Analytical precision of the Seralyzer and Dacos ALT methods using duplicate testing of control sera.*

| | Seralyzer ($n$ = 15) | | | | Dacos ($n$ = 15) | | | |
|---|---|---|---|---|---|---|---|---|
| | Replicate 1 | | Replicate 2 | | Replicate 1 | | Replicate 2 | |
| | Mean | s.d. | Mean | s.d. | Mean | s.d. | Mean | s.d. |
| Gilford N | 24·5 | 3·3 | 24·2 | 3·9 | 19·3 | 1·4 | 19·8 | 1·8 |
| Gilford A | 74·8 | 5·1 | 76·4 | 5·2 | 77·7 | 2·8 | 78·1 | 2·8 |
| Wellcome | 33·3 | 2·1 | 33·7 | 2·8 | 33·4 | 0·5 | 33·5 | 1·2 |

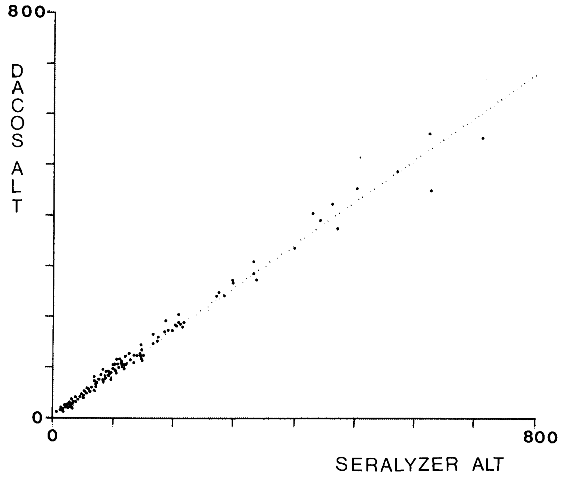

*Figure 1. Comparison of serum ALT levels (IU $l^{-1}$) between Seralyzer and Dacos analysers.*

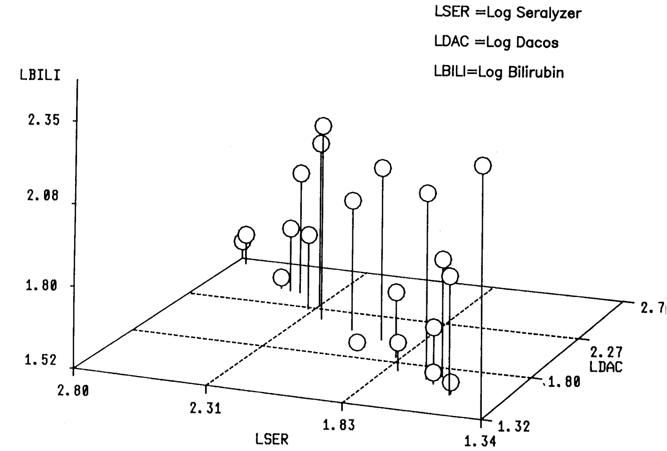

*Figure 2. Comparison of Seralyzer and Dacos ALT results for clinical samples with increased concentrations of bilirubin. (Log ALT IU $l^{-1}$ horizontal axis, log bilirubin µmol $l^{-1}$ vertical axis.)*

than the Dacos results, but the same for Wellcome serum ($p$ >0·2). The results for the Seralyzer were slightly less consistent day to day than the Dacos, but were acceptable for clinical purposes.

Analysis of duplicate Seralyzer results showed the 115 valid pairs of observations greater than 10 IU $l^{-1}$ had a mean difference of 1·03 and a standard deviation (SD) of 1·04, with a correlation coefficient of 0·99.

Comparisons of the Seralyzer and Dacos results are shown in figure 1. Calculations using ALT results with levels less than 250 IU $l^{-1}$ gave a very good correlation coefficient of 0·99 ($p$ <0·001) when comparing the Seralyzer to the Dacos method.

The slope was 0·91 and the intercept 2·0. Serum with levels of ALT greater than 250 IU $l^{-1}$, which had to be reanalysed after further dilution, showed little change in statistical characteristics up to a level of approx. 740 IU $l^{-1}$, the highest levels analysed.

Twenty samples with bilirubin concentrations greater than 40 µmol $l^{-1}$ were available for analysis (max 230 µmol $l^{-1}$). The Seralyzer and Dacos results for ALT were very similar ($p$ <0·001, $r$ = 0·99). As the Dacos method is known not to be affected by high bilirubin levels, it can be construed that bilirubin does not interfere with this reflectometric method. Figure 2 shows log ALT levels of the two methods on the $x$-axis and log bilirubin results on the $y$-axis. The logarithm of the results were used in figure 2 to give a clearer visual representation. The various levels of bilirubin show little effect on the ALT comparisons. Further evidence was obtained by adding increasing amounts of human bilirubin to serum: enzyme estimations showed no significant effect by either method, at bilirubin concentrations up to 120 µmol $l^{-1}$.

The red pigment of haemoglobin can affect reflectometric measurements. The presence of visual haemoglobin in serum depresses the level of ALT as measured by the Seralyzer. To investigate this, increasing amounts of haemoglobin were added to serum. The ALT results show a significant interference effect only when the haemoglobin concentration is greater than about 100 mg $dl^{-1}$ (figure 3). This is the approximate level at which haemoglobin becomes visible.

### Discussion

Serum ALT results showed a good correlation between the Ames Seralyzer and the standard laboratory procedure. Satisfactory results were obtained for serum samples tested with ALT levels from 10–800 IU $l^{-1}$. Increased levels of bilirubin do not affect the test.

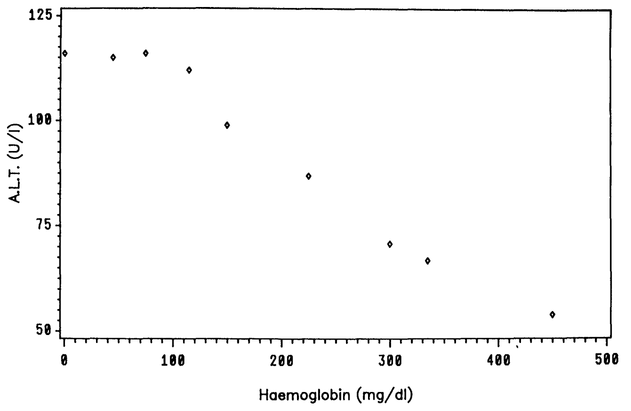

*Figure 3. Seralyzer ALT measurements on a clinical sample with added amounts of haemoglobin.*

Samples showing any visible haemolysis should not be processed.

Day-to-day imprecision for the reflectometer is acceptable for clinical purposes but was marginally greater than that for the automated laboratory analyser. One cause of imprecision may be due to small variations in the daily two-point calibrations. However, in practice, calibration would only be carried out once for each batch of strips. The analyser flags when the calibration is not within preset levels but to identify possible inaccuracies, controls at high and low concentration can be processed after each calibration, according to good laboratory practice.

Although the system is reliable and simple to use, some expertise is required for the dilution of the serum and for standardization of technique. Because of these aspects, it would operate most effectively in the laboratory environment or by well trained appropriate personnel in other clinical situations.

The Seralyzer method is particularly suited for processing small batches of urgent analyses and for high risk samples as the reaction strips are totally disposable and the machine easily decontaminated.

In conclusion, this reflectometric method for ALT is simple and convenient, and with care and suitable samples, produces clinically useful results which compare well with standard laboratory techniques.

### References

1. Zipp, A. and Hornby, W. E. Solid Phase Chemistry: its principles and application in clinical analysis. *Talanta*, **31** (1984), 863.
2. Stevens, J. F. and Newall, R. G. Application of reflectance spectroscopy to the estimation of uric acid, urea and glucose: an evaluation of the Ames Seralyzer. *Journal of Clinical Pathology*, **36** (1983), 9.
3. Stevens, J. F., Tsang, W. and Newall, R. G. Measurement of the enzymes lactate dehydrogenase and creatinine kinase using reflectance spectroscopy. *Journal of Clinical Pathology*, **36** (1983), 1371.
4. Stevens, J. F. and Newall, R. G. Measurement of serum AST activity using the Seralyzer system. *Journal of Automatic Chemistry*, **7** (1985), 204.

